# Supplementary material for: NO3− anions can act as Lewis acid in the solid state
Source: Nat Commun. 2017 Feb 21;8:14522. doi: 10.1038/ncomms14522 (PMC5321794; doi:10.1038/ncomms14522)
Supplement: Supplementary Information — Supplementary Figures, Supplementary Tables and Supplementary Notes [file ncomms14522-s1.pdf]

Supplementary Note 1

For our initial calculations the cyanamide cation seemed an ideal candidate for two reasons: its positive charge would complement the negative charge in **2** while leaving some electron density on N, and its C_3v symmetry renders the computations more economical.

As is summarized in entries 1-3 in Supplementary Table 1, the complexation energies for all three cyanamide structures are negative and fairly large (comparable to hydrogen bonding). This large enthalpy might depend significantly on simple charge compensation. As a matter of fact, the interaction energy of host **2a** with the cyanamide molecule flipped 180 degrees (so that the $–NH_3^+$ and nitrile N-atoms switch position, i.e. [**2a**···N^+H_3–C≡N], not shown in the table) is –47.7 kcal/mol. This is nearly identical to the interaction energy of complex [**2a**···N≡C–N^+H_3] (entry 1, –48.8 kcal/mol), thus confirming that the nature of the interaction is predominantly charge compensation. Interestingly, the least stable of these complexes involves **2c**, which actually had a positive potential on the nitrate's N-atom (see Figure 2b). This implies that the energy gain of charge compensation is largely counteracted by repulsion between the π-hole and the cyanamide cation.

Next we considered the charge neutral species acetonitrile and hydrogen cyanide as electron rich partners for the π-hole in **2c** (entries 4 and 5 in Supplementary Table 1). Interestingly, the estimated binding enthalpies of these 'pseudo anti-electrostatic' complexes are negative and in the order of weak hydrogen bonding (up to –7.7 kcal/mol for [**2c**···N≡CCH_3]$^–$).

Stimulated by these findings, we wondered how cyanide, chloride and $BF_4^–$ anions would interact with the π-hole in **2c** and if perhaps truly 'anti-electrostatic' complexes might be obtained. To our surprise these complexes (entries 6, 7 and 8 in Supplementary Table 1) converged with their initial C_3v symmetry intact to give quite large and negative energies: –20.7 kcal/mol for [**2c**···NC]$^{2–}$, –31.6 kcal/mol for [**2c**···Cl]$^{2–}$ and –8.2 kcal/mol for [**2c**···BF_4]$^{2–}$. Such enthalpies are comparable to medium to strong hydrogen bonding. Unsurprisingly, when considering **2a** as partner to these anions (entries 9, 10 and 11 in Supplementary Table 1), the computed enthalpies were fairly large but positive.

An 'atoms-in-molecules' analysis of all complexes (see Supplementary Figures 1 and 2) indicate a single bond critical point between the nitrate's N-atom and the interacting electronegative atom.

Supplementary Table 1. Overview of some characteristics of complexes involving **2** with C_3v symmetry as computed with the DFT paradigm at the BP86-D3/def2-TZVP level of theory.

Entry	Host	Guest	ΔE (kcal/mol)	Distance (Å)	10^2 x ρ (a.u.)
1	**2a**	$^{+}NH_3CN$	-48.8	2.719	1.28
2	**2b**		-40.2	2.783	1.11
3	**2c**		-22.4	2.972	0.73
4		CH_3CN	-7.7	2.867	1.00
5		HCN	-5.7	2.920	0.89
6		$N{\equiv}C^{-}$	-20.7	2.649	1.70
7		Cl^{-}	-31.6	2.943	1.43
8		BF_4^{-}	-8.2	2.472	1.75
9	**2a**	$N{\equiv}C^{-}$	+22.3	2.728	1.45
10		Cl^{-}	+14.7	2.997	1.28
11		BF_4^{-}	+28.6	2.604	1.27

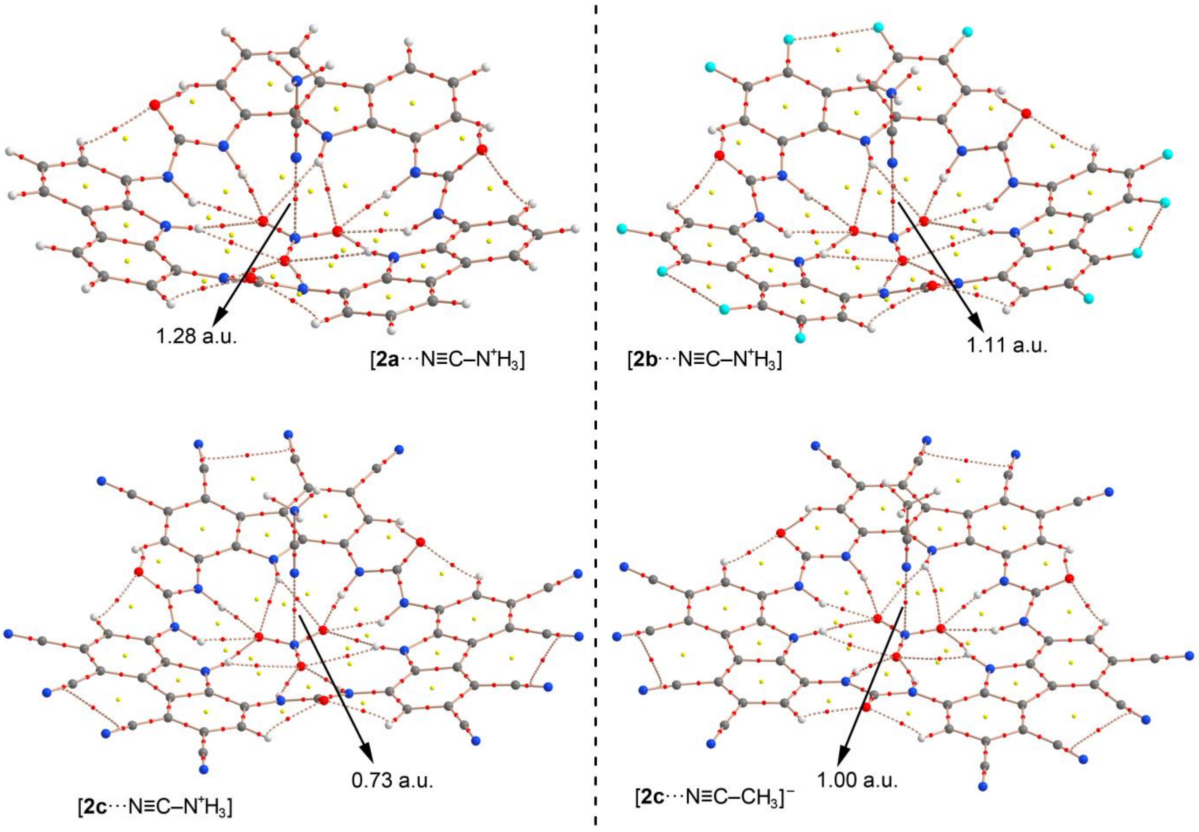

Supplementary Figure 1. Renderings of 'atoms-in-molecules' analyses of the complexes corresponding to entries **1** to **4** (Table S1). The charge density values (10^2 x ρ, a.u.) at the bond CPs that characterize the π-hole interaction are given.

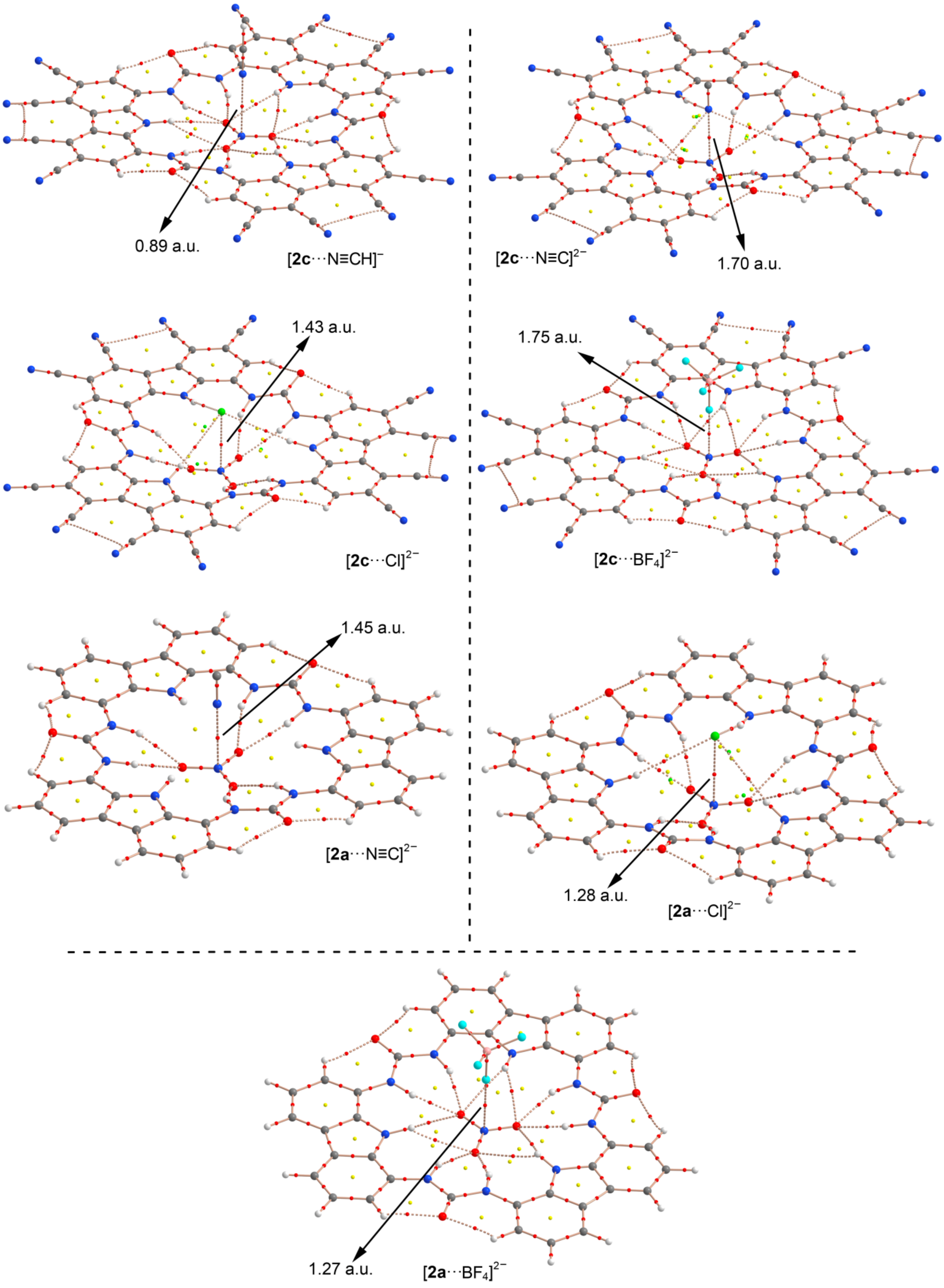

Supplementary Figure 2. Renderings of ‘atoms-in-molecules’ analyses of the complexes corresponding to entries **5** to **11** (Table S1). The charge density values (10^2 x ρ, a.u.) at the bond CPs that characterize the π-hole interaction are given.

Supplementary Table 2. *Numerical overview of the CSD and PDB data.*

DB:	CU:	Partner:	$N_{struct.}$	N_{total}	$N_{r≤1}$	$N_{≤vdW@r≤1}$	N_{r3-4}	$N_{vdW@r≤3-4}$
PDB	NO_3	N···O=C	1,560	20,058	1,296	266 (20.5%)	5,940	1,180 (19.9%)
PDB	NO_3	N···OH_2	1,437	24,218	430	86 (19.5%)	12,086	7,312 (60.5%)
PDB	NO_3	N···SC	120	840	168	36 (21.4%)		
PDB	NO_3	N···SCC	56	354	48	0 (0.0%)		
CSD	NO_3	N···ElR	7,995	117,638[a]	5,540	494 (8.9%)	47,850	28 (0.06%)
CSD	NO_3	N···O=X	5,787	26,364	1,937	327 (16.9%)		
CSD	NO_3	N···OH_2	2,649	10,526	91	11 (12.0%)	7,406	2 (0.03%)
CSD	NO_3	N···S	732	2,139	167	22 (13.2%)		

a) 'ElR' stands for 'electron rich and the data consists of ElR = N (89,845), O (53,504), F (433), P (470), S (2,071), Cl (999), As (33), Se (33), Br (186), Te (21) and I (43).

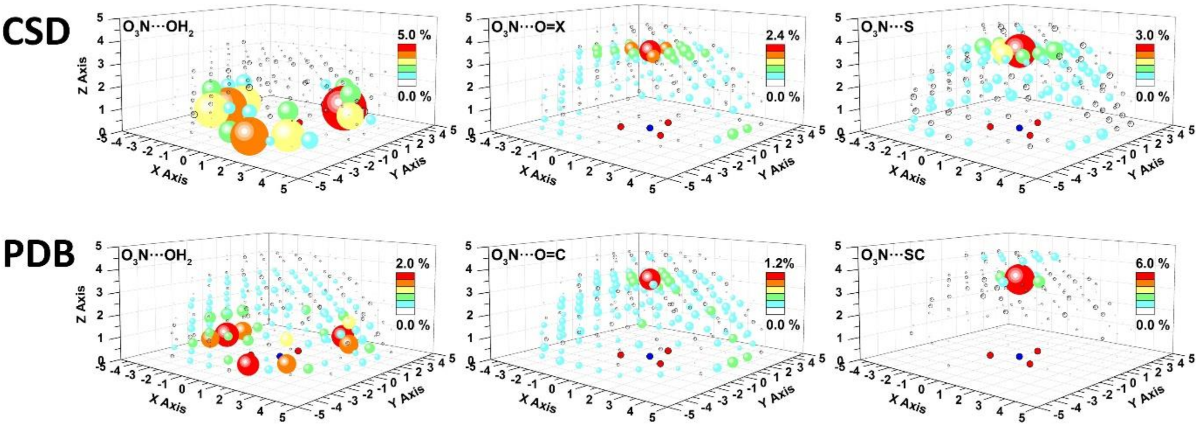

Supplementary Figure 3. Four dimensional (4D) density plots of the CSD/PDB data found within the 10 Å wide and 5 Å high hemisphere.

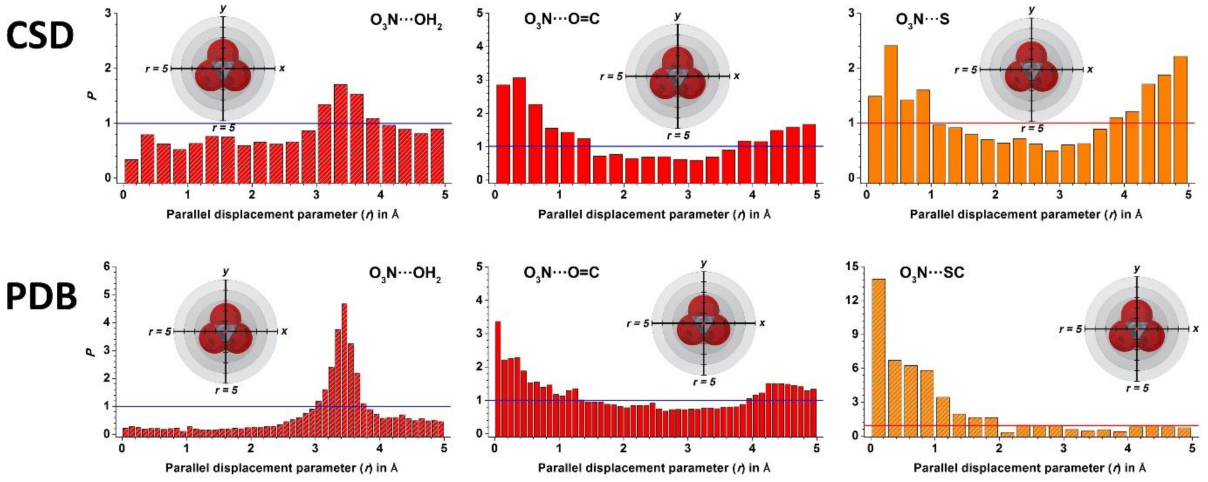

Supplementary Figure 4. Directionality plots *P*(*r*) of the data CSD/PDB data found within the 10 Å wide and 5 Å high hemisphere.

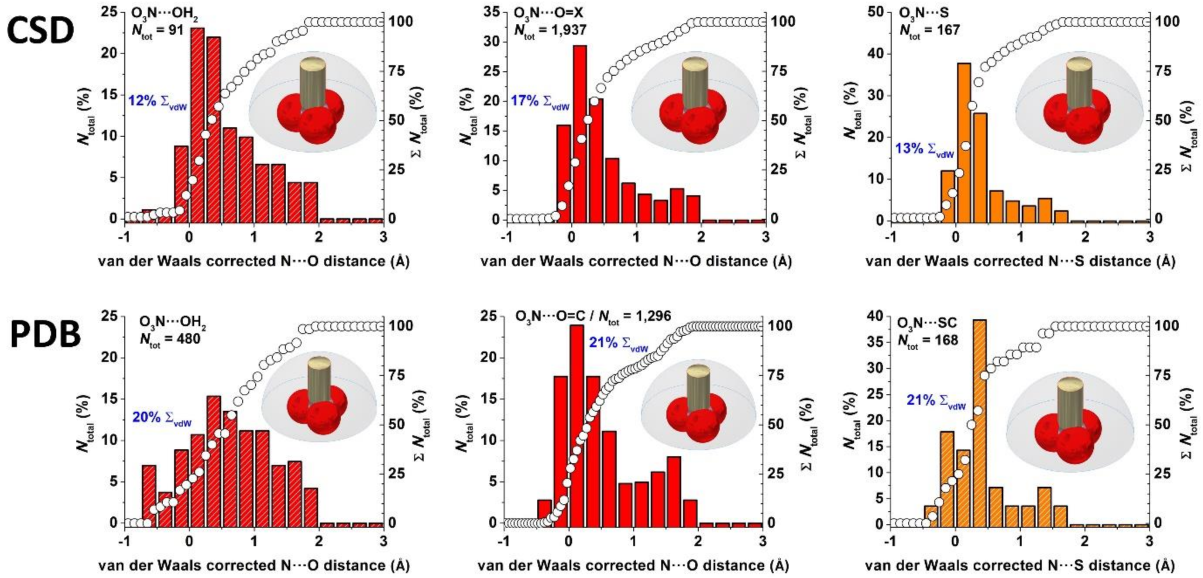

Supplementary Figure 5. Distribution of the van der Waals corrected $^{nitrate}N \cdots O/S$ distances within a spherical segment with base radius of 1 Å located within the 10 Å wide and 5 Å high hemisphere studied herein (gold in inset figures).

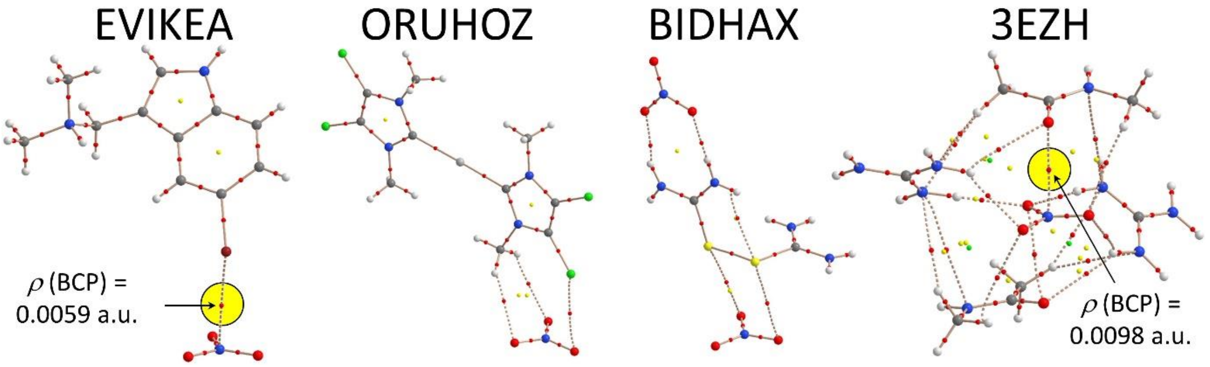

Supplementary Figure 6. Renderings of 'atoms-in-molecules' analyses of the computed fragments of EVIKEA, ORUHOZ and BIDHAX (see also Figure 4).

Supplementary Note 2

The Natural Bond Orbital analysis (with a focus on second order perturbation) revealed the following donor-acceptor interactions:

EVIKEA, LP_{Br}→LP^*_N, $E^{(2)}$ = 0.51 kcal/mol;

ORUHOZ, LP_{Cl}→π^*_{NO3}, $E^{(2)}$ = 0.24 kcal/mol;

BIDHAX, LP_S→π^*_{NO3}, $E^{(2)}$ = 0.12 kcal/mol, $LP_{S\text{–}S}$→Ry^*_N, $E^{(2)}$ = 0.24 kcal/mol;

3EZH, $LP_{C=O}$→π^*_{NO3}, $E^{(2)}$ = 0.34 kcal/mol, $LP_{C=O}$→Ry^*_N, $E^{(2)}$ = 0.13 kcal/mol.

Supplementary Note 3

After some deliberation we could not think of a single (common) anion with the combined properties of NO_3^-, i.e.: fairly polarized and further polarizable, not so charge-dense, and flat, i.e. π-hole is sterically accessible. The halides are easily dismissed (point charge) and most other common anions are not flat and sterically too crowded to allow the approach of a Lewis base (e.g. ClO_3^-, BrO_3^-, ClO_4^-, BrO_4^-, BF_4^-, PF_6^-). Anions with two or more charges such as CO_3^{2-} are likely too charge dense. Flat anions such as NO_2^-, N_3^- and CN^- do not seem to be polarized enough and thus also too charge dense, i.e. the maximum potentials are respectively is –132, –142 and –145 kcal/mol versus –110 kcal/mol for NO_3^- (MP2/6-311+G**). They are also intrinsically more charge dense with an atom over charge ratio of 3 (NO_2^-, N_3^-) or 2 (CN^-) versus 4 of nitrate. We also contemplated trifluoroacetate but the maximum potentials (maximum actually lies on the σ-hole of -CF_3) are sterically less accessible. A promising candidate might be the rare FCO_2^- (similar MEP as nitrate, only one structure in the CSD and none in the PDB), but this anion is very likely to decompose to F^- and CO_2. I_3^- (and possibly Hlg_3^- generally) can act as a halogen bond donor but is also prone to decomposition into I^- and I_2 (P. H. Svensson and L. Kloo, *Chemical Reviews*, **2003**, *103*, 1649-1684; at the MP2/6-311+G** level of theory two latent σ-holes are present on I_3^- (–76 kcal/mol), Br_3^- (–87 kcal/mol) and Cl_3^- (–97 kcal/mol)).

Supplementary Table 3. *Cartesian coordinates of computed complexes.*

2a

C	5.3638114	-3.4363870	0.0000000
C	6.7085655	-3.0242613	0.0000000
C	7.0650630	-1.6800364	0.0000000
C	6.0395387	-0.7231499	0.0000000
C	4.6822073	-1.1351474	0.0000000
C	4.3188586	-2.5014614	0.0000000
C	6.0395387	0.7231499	0.0000000
C	4.6822073	1.1351474	0.0000000
N	3.8730678	0.0000000	0.0000000
C	7.0650630	1.6800364	0.0000000
C	6.7085655	3.0242613	0.0000000
C	5.3638114	3.4363870	0.0000000
C	4.3188586	2.5014614	0.0000000
N	2.9544491	2.8229008	0.0000000
C	2.3567360	4.0819866	0.0000000
O	2.9706882	5.1453828	0.0000000
N	0.9674792	3.9700784	0.0000000
H	5.1096314	-4.4921733	0.0000000
H	7.4841208	-3.7917815	0.0000000
H	8.1111494	-1.3716519	0.0000000
H	2.8570966	0.0000000	0.0000000
H	8.1111494	1.3716519	0.0000000
H	7.4841208	3.7917815	0.0000000
H	5.1096314	4.4921733	0.0000000
H	2.3168711	2.0303412	0.0000000
H	0.5998915	3.0216398	0.0000000
C	0.2940927	6.3633904	0.0000000
C	-0.7351956	7.3219189	0.0000000
C	-2.0775773	6.9585423	0.0000000
C	-2.3935031	5.5919689	0.0000000
C	-1.3580372	4.6224841	0.0000000
C	0.0068998	4.9909720	0.0000000
C	-3.6460356	4.8688190	0.0000000
C	-3.3241701	3.4873367	0.0000000
N	-1.9365339	3.3541751	0.0000000
C	-4.9874857	5.2785058	0.0000000
C	-5.9733699	4.2976575	0.0000000
C	-5.6579041	2.9270035	0.0000000
C	-4.3257584	2.4895106	0.0000000
N	-3.9219284	1.1471776	0.0000000
C	-4.7134721	0.0000000	0.0000000
O	-5.9413763	0.0000000	0.0000000
N	-3.9219284	-1.1471776	0.0000000
H	1.3355205	6.6711572	0.0000000

H	-0.4582813	8.3773295	0.0000000
H	-2.8676893	7.7102874	0.0000000
H	-1.4285483	2.4743183	0.0000000
H	-5.2434601	6.3386355	0.0000000
H	-7.0258395	4.5855479	0.0000000
H	-6.4451519	2.1789839	0.0000000
H	-2.9167626	0.9912986	0.0000000
H	-2.9167626	-0.9912986	0.0000000
C	-5.6579041	-2.9270035	0.0000000
C	-5.9733699	-4.2976575	0.0000000
C	-4.9874857	-5.2785058	0.0000000
C	-3.6460356	-4.8688190	0.0000000
C	-3.3241701	-3.4873367	0.0000000
C	-4.3257584	-2.4895106	0.0000000
C	-2.3935031	-5.5919689	0.0000000
C	-1.3580372	-4.6224841	0.0000000
N	-1.9365339	-3.3541751	0.0000000
C	-2.0775773	-6.9585423	0.0000000
C	-0.7351956	-7.3219189	0.0000000
C	0.2940927	-6.3633904	0.0000000
C	0.0068998	-4.9909720	0.0000000
N	0.9674792	-3.9700784	0.0000000
C	2.3567360	-4.0819866	0.0000000
O	2.9706882	-5.1453828	0.0000000
N	2.9544491	-2.8229008	0.0000000
H	-6.4451519	-2.1789839	0.0000000
H	-7.0258395	-4.5855479	0.0000000
H	-5.2434601	-6.3386355	0.0000000
H	-1.4285483	-2.4743183	0.0000000
H	-2.8676893	-7.7102874	0.0000000
H	-0.4582813	-8.3773295	0.0000000
H	1.3355205	-6.6711572	0.0000000
H	0.5998915	-3.0216398	0.0000000
H	2.3168711	-2.0303412	0.0000000
N	-0.0000000	0.0000000	0.0000000
O	0.6299835	1.0911634	0.0010000
O	0.6299835	-1.0911634	0.0010000
O	-1.2599670	0.0000000	0.0010000

2b

C	5.3390898	-3.4461727	0.0000000
C	6.6752365	-3.0382865	0.0000000
C	7.0527565	-1.7020898	0.0000000
C	6.0503601	-0.7224723	0.0000000
C	4.6897754	-1.1360228	0.0000000
C	4.3106108	-2.4974785	0.0000000
C	6.0503601	0.7224723	0.0000000

C	4.6897754	1.1360228	0.0000000
N	3.8859208	0.0000000	0.0000000
C	7.0527565	1.7020898	0.0000000
C	6.6752365	3.0382865	0.0000000
C	5.3390898	3.4461727	0.0000000
C	4.3106108	2.4974785	0.0000000
N	2.9455959	2.8084565	0.0000000
C	2.3478163	4.0665370	0.0000000
O	2.9615581	5.1295691	0.0000000
N	0.9593967	3.9551891	0.0000000
H	5.1044272	-4.5066372	0.0000000
H	2.8701710	0.0000000	0.0000000
H	5.1044272	4.5066372	0.0000000
H	2.3109717	2.0136731	0.0000000
H	0.5884062	3.0081968	0.0000000
C	0.3149282	6.3468737	0.0000000
C	-0.7063850	7.3000676	0.0000000
C	-2.0523252	6.9589112	0.0000000
C	-2.3995007	5.6010017	0.0000000
C	-1.3610631	4.6294760	0.0000000
C	0.0075744	4.9818377	0.0000000
C	-3.6508594	4.8785294	0.0000000
C	-3.3287123	3.4934533	0.0000000
N	-1.9429604	3.3653062	0.0000000
C	-5.0004313	5.2568214	0.0000000
C	-5.9688515	4.2617811	0.0000000
C	-5.6540180	2.9007010	0.0000000
C	-4.3181852	2.4843592	0.0000000
N	-3.9049926	1.1467326	0.0000000
C	-4.6956325	0.0000000	0.0000000
O	-5.9231162	0.0000000	0.0000000
N	-3.9049926	-1.1467326	0.0000000
H	1.3506487	6.6738822	0.0000000
H	-1.4350855	2.4856410	0.0000000
H	-6.4550759	2.1672450	0.0000000
H	-2.8993779	0.9945237	0.0000000
H	-2.8993779	-0.9945237	0.0000000
C	-5.6540180	-2.9007010	0.0000000
C	-5.9688515	-4.2617811	0.0000000
C	-5.0004313	-5.2568214	0.0000000
C	-3.6508594	-4.8785294	0.0000000
C	-3.3287123	-3.4934533	0.0000000
C	-4.3181852	-2.4843592	0.0000000
C	-2.3995007	-5.6010017	0.0000000
C	-1.3610631	-4.6294760	0.0000000
N	-1.9429604	-3.3653062	0.0000000
C	-2.0523252	-6.9589112	0.0000000

C	-0.7063850	-7.3000676	0.0000000
C	0.3149282	-6.3468737	0.0000000
C	0.0075744	-4.9818377	0.0000000
N	0.9593967	-3.9551891	0.0000000
C	2.3478163	-4.0665370	0.0000000
O	2.9615581	-5.1295691	0.0000000
N	2.9455959	-2.8084565	0.0000000
H	-6.4550759	-2.1672450	0.0000000
H	-1.4350855	-2.4856410	0.0000000
H	1.3506487	-6.6738822	0.0000000
H	0.5884062	-3.0081968	0.0000000
H	2.3109717	-2.0136731	0.0000000
N	-0.0000000	0.0000000	0.0000000
O	0.6297742	1.0908009	0.0000000
O	0.6297742	-1.0908009	0.0000000
O	-1.2595483	0.0000000	0.0000000
F	7.6450679	3.9892877	0.0000000
F	8.3620686	1.3736855	0.0000000
F	8.3620686	-1.3736855	0.0000000
F	7.6450679	-3.9892877	0.0000000
F	-0.3677095	-8.6154669	0.0000000
F	-2.9913877	-7.9286066	0.0000000
F	-5.3706809	-6.5549211	0.0000000
F	-7.2773584	-4.6261792	0.0000000
F	-7.2773584	4.6261792	0.0000000
F	-5.3706809	6.5549211	0.0000000
F	-2.9913877	7.9286066	0.0000000
F	-0.3677095	8.6154669	0.0000000

2c

C	5.2939769	-3.4547115	0.0000000
C	6.6514832	-3.0917641	0.0000000
C	7.0809109	-1.7407868	0.0000000
C	6.0858125	-0.7296262	0.0000000
C	4.7177623	-1.1304292	0.0000000
C	4.2945870	-2.4816845	0.0000000
C	6.0858125	0.7296262	0.0000000
C	4.7177623	1.1304292	0.0000000
N	3.9195503	0.0000000	0.0000000
C	7.0809109	1.7407868	0.0000000
C	6.6514832	3.0917641	0.0000000
C	5.2939769	3.4547115	0.0000000
C	4.2945870	2.4816845	0.0000000
N	2.9279431	2.7637521	0.0000000
C	2.3238589	4.0250417	0.0000000
O	2.9346637	5.0829866	0.0000000
N	0.9295080	3.9175491	0.0000000

H	5.0196796	-4.5043806	0.0000000
H	2.9034588	0.0000000	0.0000000
H	5.0196796	4.5043806	0.0000000
H	2.2971056	1.9650605	0.0000000
H	0.5532395	2.9718821	0.0000000
C	0.3448794	6.3120743	0.0000000
C	-0.6481953	7.3062355	0.0000000
C	-2.0328899	7.0026422	0.0000000
C	-2.4110314	5.6352814	0.0000000
C	-1.3799008	4.6509166	0.0000000
C	0.0019084	4.9600637	0.0000000
C	-3.6747811	4.9056551	0.0000000
C	-3.3378616	3.5204874	0.0000000
N	-1.9597752	3.3944302	0.0000000
C	-5.0480211	5.2618553	0.0000000
C	-6.0032879	4.2144714	0.0000000
C	-5.6388564	2.8573628	0.0000000
C	-4.2964954	2.4783792	0.0000000
N	-3.8574511	1.1537970	0.0000000
C	-4.6477179	0.0000000	0.0000000
O	-5.8693274	0.0000000	0.0000000
N	-3.8574511	-1.1537970	0.0000000
H	1.3910683	6.5993603	0.0000000
H	-1.4517294	2.5144691	0.0000000
H	-6.4107478	2.0949797	0.0000000
H	-2.8503451	1.0068216	0.0000000
H	-2.8503451	-1.0068216	0.0000000
C	-5.6388564	-2.8573628	0.0000000
C	-6.0032879	-4.2144714	0.0000000
C	-5.0480211	-5.2618553	0.0000000
C	-3.6747811	-4.9056551	0.0000000
C	-3.3378616	-3.5204874	0.0000000
C	-4.2964954	-2.4783792	0.0000000
C	-2.4110314	-5.6352814	0.0000000
C	-1.3799008	-4.6509166	0.0000000
N	-1.9597752	-3.3944302	0.0000000
C	-2.0328899	-7.0026422	0.0000000
C	-0.6481953	-7.3062355	0.0000000
C	0.3448794	-6.3120743	0.0000000
C	0.0019084	-4.9600637	0.0000000
N	0.9295080	-3.9175491	0.0000000
C	2.3238589	-4.0250417	0.0000000
O	2.9346637	-5.0829866	0.0000000
N	2.9279431	-2.7637521	0.0000000
H	-6.4107478	-2.0949797	0.0000000
H	-1.4517294	-2.5144691	0.0000000
H	1.3910683	-6.5993603	0.0000000

H	0.5532395	-2.9718821	0.0000000
H	2.2971056	-1.9650605	0.0000000
N	-0.0000000	0.0000000	0.0000000
O	0.6290045	1.0894678	0.0000000
O	0.6290045	-1.0894678	0.0000000
O	-1.2580091	0.0000000	0.0000000
C	7.6117718	4.1508676	0.0000000
C	8.4890263	1.5371675	0.0000000
C	8.4890263	-1.5371675	0.0000000
C	7.6117718	-4.1508676	0.0000000
C	-0.2111291	-8.6674215	0.0000000
C	-2.9132870	-8.1202962	0.0000000
C	-5.5757393	-6.5831286	0.0000000
C	-7.4006427	-4.5165540	0.0000000
C	-7.4006427	4.5165540	0.0000000
C	-5.5757393	6.5831286	0.0000000
C	-2.9132870	8.1202962	0.0000000
C	-0.2111291	8.6674215	0.0000000
N	0.1886199	-9.7617700	0.0000000
N	-3.5069753	-9.1233231	0.0000000
N	-6.1475419	-7.5987913	0.0000000
N	-8.5482508	-4.7175354	0.0000000
N	-8.5482508	4.7175354	0.0000000
N	-6.1475419	7.5987913	0.0000000
N	-3.5069753	9.1233231	0.0000000
N	0.1886199	9.7617700	0.0000000
N	8.3596309	5.0442346	0.0000000
N	9.6545172	1.5245318	0.0000000
N	9.6545172	-1.5245318	0.0000000
N	8.3596309	-5.0442346	0.0000000

[**2a**···N≡C–N^+H_3]

C	-5.2914303	3.4327112	-0.1394568
C	-6.6081970	3.0333374	0.1462972
C	-6.9714754	1.6892419	0.1981552
C	-5.9831730	0.7236951	-0.0363193
C	-4.6520885	1.1309190	-0.3151502
C	-4.2854641	2.4862797	-0.3741903
C	-5.9831730	-0.7236951	-0.0363193
C	-4.6520885	-1.1309190	-0.3151502
N	-3.8561606	0.0000000	-0.4760614
C	-6.9714754	-1.6892419	0.1981552
C	-6.6081970	-3.0333374	0.1462972
C	-5.2914303	-3.4327112	-0.1394568
C	-4.2854641	-2.4862797	-0.3741903
N	-2.9516064	-2.8094672	-0.6751942
C	-2.2704660	-3.9325624	-0.2179864

O	-2.7651713	-4.7894172	0.5077253
N	-0.9572668	-3.9608997	-0.6751942
H	-5.0329564	4.4875758	-0.1659894
H	-7.3590329	3.8041811	0.3232851
H	-7.9972332	1.3919154	0.4176196
H	-2.8678459	0.0000000	-0.7177057
H	-7.9972332	-1.3919154	0.4176196
H	-7.3590329	-3.8041811	0.3232851
H	-5.0329564	-4.4875758	-0.1659894
H	-2.4118256	-2.1140801	-1.1893006
H	-0.6249343	-3.1457423	-1.1893006
C	-0.3271000	-6.2988686	-0.1394568
C	0.6771512	-7.2395352	0.1462972
C	2.0228113	-6.8820957	0.1981552
C	2.3648482	-5.5434274	-0.0363193
C	1.3466397	-4.5942863	-0.3151502
C	-0.0104493	-4.9544607	-0.3741903
C	3.6183248	-4.8197323	-0.0363193
C	3.3054488	-3.4633673	-0.3151502
N	1.9280803	-3.3395331	-0.4760614
C	4.9486641	-5.1928539	0.1981552
C	5.9310458	-4.2061978	0.1462972
C	5.6185302	-2.8661574	-0.1394568
C	4.2959135	-2.4681810	-0.3741903
N	3.9088731	-1.1514325	-0.6751942
C	4.5409319	0.0000000	-0.2179864
O	5.5303426	0.0000000	0.5077253
N	3.9088731	1.1514325	-0.6751942
H	-1.3698764	-6.6024560	-0.1659894
H	0.3849990	-8.2752000	0.3232851
H	2.7931825	-7.6217648	0.4176196
H	1.4339229	-2.4836274	-0.7177057
H	5.2040507	-6.2298494	0.4176196
H	6.9740339	-4.4710189	0.3232851
H	6.4028329	-2.1148802	-0.1659894
H	3.0367599	-1.0316622	-1.1893006
H	3.0367599	1.0316622	-1.1893006
C	5.6185302	2.8661574	-0.1394568
C	5.9310458	4.2061978	0.1462972
C	4.9486641	5.1928539	0.1981552
C	3.6183248	4.8197323	-0.0363193
C	3.3054488	3.4633673	-0.3151502
C	4.2959135	2.4681810	-0.3741903
C	2.3648482	5.5434274	-0.0363193
C	1.3466397	4.5942863	-0.3151502
N	1.9280803	3.3395331	-0.4760614
C	2.0228113	6.8820957	0.1981552

C	0.6771512	7.2395352	0.1462972
C	-0.3271000	6.2988686	-0.1394568
C	-0.0104493	4.9544607	-0.3741903
N	-0.9572668	3.9608997	-0.6751942
C	-2.2704660	3.9325624	-0.2179864
O	-2.7651713	4.7894172	0.5077253
N	-2.9516064	2.8094672	-0.6751942
H	6.4028329	2.1148802	-0.1659894
H	6.9740339	4.4710189	0.3232851
H	5.2040507	6.2298494	0.4176196
H	1.4339229	2.4836274	-0.7177057
H	2.7931825	7.6217648	0.4176196
H	0.3849990	8.2752000	0.3232851
H	-1.3698764	6.6024560	-0.1659894
H	-0.6249343	3.1457423	-1.1893006
H	-2.4118256	2.1140801	-1.1893006
N	0.0000000	0.0000000	-1.5338053
O	-0.6314494	-1.0937024	-1.5199062
O	-0.6314494	1.0937024	-1.5199062
O	1.2628987	0.0000000	-1.5199062
N	0.0000000	0.0000000	1.1850118
C	0.0000000	0.0000000	2.3452413
H	-0.4881515	0.8455031	4.1267668
H	-0.4881515	-0.8455031	4.1267668
H	0.9763029	0.0000000	4.1267668
N	0.0000000	0.0000000	3.7565177

[**2b**···N≡C–N$^+$H$_3$]

C	5.2604226	-3.4418179	-0.1550882
C	6.5641499	-3.0472654	0.1499425
C	6.9497318	-1.7099973	0.1990322
C	5.9883832	-0.7230505	-0.0529820
C	4.6577099	-1.1318226	-0.3481464
C	4.2757028	-2.4823367	-0.4106593
C	5.9883832	0.7230505	-0.0529820
C	4.6577099	1.1318226	-0.3481464
N	3.8721548	0.0000000	-0.5311224
C	6.9497318	1.7099973	0.1990322
C	6.5641499	3.0472654	0.1499425
C	5.2604226	3.4418179	-0.1550882
C	4.2757028	2.4823367	-0.4106593
N	2.9454124	2.7964593	-0.7276229
C	2.2602787	3.9149175	-0.2667166
O	2.7536475	4.7694574	0.4621726
N	0.9490986	3.9490316	-0.7276229
H	5.0224059	-4.5012146	-0.1713131
H	2.8713044	0.0000000	-0.7146953

H	5.0224059	4.5012146	-0.1713131
H	2.4136532	2.1001980	-1.2486121
H	0.6119982	3.1403840	-1.2486121
C	0.3504905	6.2765686	-0.1550882
C	-0.6430657	7.2083533	0.1499425
C	-1.9939648	6.8736430	0.1990322
C	-2.3680115	5.5476173	-0.0529820
C	-1.3486679	4.5996064	-0.3481464
C	0.0119152	4.9440356	-0.4106593
C	-3.6203718	4.8245667	-0.0529820
C	-3.3090420	3.4677838	-0.3481464
N	-1.9360774	3.3533844	-0.5311224
C	-4.9557670	5.1636456	0.1990322
C	-5.9210842	4.1610879	0.1499425
C	-5.6109131	2.8347506	-0.1550882
C	-4.2876180	2.4616989	-0.4106593
N	-3.8945110	1.1525723	-0.7276229
C	-4.5205574	0.0000000	-0.2667166
O	-5.5072950	0.0000000	0.4621726
N	-3.8945110	-1.1525723	-0.7276229
H	1.3869633	6.6001384	-0.1713131
H	-1.4356522	2.4866226	-0.7146953
H	-6.4093692	2.0989238	-0.1713131
H	-3.0256514	1.0401860	-1.2486121
H	-3.0256514	-1.0401860	-1.2486121
C	-5.6109131	-2.8347506	-0.1550882
C	-5.9210842	-4.1610879	0.1499425
C	-4.9557670	-5.1636456	0.1990322
C	-3.6203718	-4.8245667	-0.0529820
C	-3.3090420	-3.4677838	-0.3481464
C	-4.2876180	-2.4616989	-0.4106593
C	-2.3680115	-5.5476173	-0.0529820
C	-1.3486679	-4.5996064	-0.3481464
N	-1.9360774	-3.3533844	-0.5311224
C	-1.9939648	-6.8736430	0.1990322
C	-0.6430657	-7.2083533	0.1499425
C	0.3504905	-6.2765686	-0.1550882
C	0.0119152	-4.9440356	-0.4106593
N	0.9490986	-3.9490316	-0.7276229
C	2.2602787	-3.9149175	-0.2667166
O	2.7536475	-4.7694574	0.4621726
N	2.9454124	-2.7964593	-0.7276229
H	-6.4093692	-2.0989238	-0.1713131
H	-1.4356522	-2.4866226	-0.7146953
H	1.3869633	-6.6001384	-0.1713131
H	0.6119982	-3.1403840	-1.2486121
H	2.4136532	-2.1001980	-1.2486121

N	-0.0000000	0.0000000	-1.4987101
O	0.6312031	1.0932758	-1.4849628
O	0.6312031	-1.0932758	-1.4849628
O	-1.2624061	0.0000000	-1.4849628
N	0.0000000	0.0000000	1.2845535
C	-0.0000000	0.0000000	2.4458292
H	0.4878124	-0.8449159	4.2299418
H	0.4878124	0.8449159	4.2299418
H	-0.9756249	0.0000000	4.2299418
N	0.0000000	0.0000000	3.8575175
F	-7.2104611	4.4854558	0.4050723
F	-5.3193348	6.4243513	0.4982037
F	-2.9039840	7.8188547	0.4982037
F	-0.2792881	8.4871704	0.4050723
F	7.4897492	4.0017146	0.4050723
F	8.2233188	1.3945034	0.4982037
F	8.2233188	-1.3945034	0.4982037
F	7.4897492	-4.0017146	0.4050723
F	-0.2792881	-8.4871704	0.4050723
F	-2.9039840	-7.8188547	0.4982037
F	-5.3193348	-6.4243513	0.4982037
F	-7.2104611	-4.4854558	0.4050723

[**2c**···N≡C–$N^{+}H_3$]

C	-5.2423712	3.4517057	-0.2655908
C	-6.5858042	3.0988221	-0.0508336
C	-7.0206679	1.7484959	-0.0108124
C	-6.0499881	0.7302645	-0.1864004
C	-4.6971235	1.1270055	-0.3960129
C	-4.2712408	2.4691261	-0.4435983
C	-6.0499881	-0.7302645	-0.1864004
C	-4.6971235	-1.1270055	-0.3960129
N	-3.9064205	0.0000000	-0.5270557
C	-7.0206679	-1.7484959	-0.0108124
C	-6.5858042	-3.0988221	-0.0508336
C	-5.2423712	-3.4517057	-0.2655908
C	-4.2712408	-2.4691261	-0.4435983
N	-2.9198748	-2.7455653	-0.6763554
C	-2.2507150	-3.8983528	-0.2634709
O	-2.7655147	-4.7900120	0.3952714
N	-0.9177919	-3.9014684	-0.6763554
H	-4.9652152	4.5010924	-0.2846463
H	-2.8931237	0.0000000	-0.6195484
H	-4.9652152	-4.5010924	-0.2846463
H	-2.3679791	-2.0265276	-1.1427950
H	-0.5710348	-3.0639939	-1.1427950
C	-0.3680792	-6.2658794	-0.2655908

C	0.6092435	-7.2528848	-0.0508336
C	1.9960921	-6.9543247	-0.0108124
C	2.3925665	-5.6045757	-0.1864004
C	1.3725463	-4.6313310	-0.3960129
C	-0.0027056	-4.9335661	-0.4435983
C	3.6574217	-4.8743112	-0.1864004
C	3.3245771	-3.5043255	-0.3960129
N	1.9532102	-3.3830594	-0.5270557
C	5.0245759	-5.2058288	-0.0108124
C	5.9765608	-4.1540627	-0.0508336
C	5.6104504	-2.8141738	-0.2655908
C	4.2739463	-2.4644399	-0.4435983
N	3.8376667	-1.1559031	-0.6763554
C	4.5014301	0.0000000	-0.2634709
O	5.5310294	0.0000000	0.3952714
N	3.8376667	1.1559031	-0.6763554
H	-1.4154528	-6.5505487	-0.2846463
H	1.4465619	-2.5055186	-0.6195484
H	6.3806680	-2.0494563	-0.2846463
H	2.9390139	-1.0374662	-1.1427950
H	2.9390139	1.0374662	-1.1427950
C	5.6104504	2.8141738	-0.2655908
C	5.9765608	4.1540627	-0.0508336
C	5.0245759	5.2058288	-0.0108124
C	3.6574217	4.8743112	-0.1864004
C	3.3245771	3.5043255	-0.3960129
C	4.2739463	2.4644399	-0.4435983
C	2.3925665	5.6045757	-0.1864004
C	1.3725463	4.6313310	-0.3960129
N	1.9532102	3.3830594	-0.5270557
C	1.9960921	6.9543247	-0.0108124
C	0.6092435	7.2528848	-0.0508336
C	-0.3680792	6.2658794	-0.2655908
C	-0.0027056	4.9335661	-0.4435983
N	-0.9177919	3.9014684	-0.6763554
C	-2.2507150	3.8983528	-0.2634709
O	-2.7655147	4.7900120	0.3952714
N	-2.9198748	2.7455653	-0.6763554
H	6.3806680	2.0494563	-0.2846463
H	1.4465619	2.5055186	-0.6195484
H	-1.4154528	6.5505487	-0.2846463
H	-0.5710348	3.0639939	-1.1427950
H	-2.3679791	2.0265276	-1.1427950
N	0.0000000	0.0000000	-1.2577631
O	-0.6300149	-1.0912177	-1.2455304
O	-0.6300149	1.0912177	-1.2455304
O	1.2600297	0.0000000	-1.2455304

C	-7.5265475	-4.1604895	0.1224557
C	-8.4115256	-1.5465503	0.2104370
C	-8.4115256	1.5465503	0.2104370
C	-7.5265475	4.1604895	0.1224557
C	0.1601841	8.5984261	0.1224557
C	2.8664109	8.0578700	0.2104370
C	5.5451146	6.5113197	0.2104370
C	7.3663634	4.4379366	0.1224557
C	7.3663634	-4.4379366	0.1224557
C	5.5451146	-6.5113197	0.2104370
C	2.8664109	-8.0578700	0.2104370
C	0.1601841	-8.5984261	0.1224557
N	-0.2499313	9.6800070	0.2602942
N	3.4590536	9.0435617	0.3974144
N	6.1024274	7.5174091	0.3974144
N	8.5080976	4.6235567	0.2602942
N	8.5080976	-4.6235567	0.2602942
N	6.1024274	-7.5174091	0.3974144
N	3.4590536	-9.0435617	0.3974144
N	-0.2499313	-9.6800070	0.2602942
N	-8.2581664	-5.0564503	0.2602942
N	-9.5614810	-1.5261525	0.3974144
N	-9.5614810	1.5261525	0.3974144
N	-8.2581664	5.0564503	0.2602942
N	-0.0000000	0.0000000	1.7136921
C	0.0000000	0.0000000	2.8729239
H	0.9737030	0.0000000	4.6555458
H	-0.4868515	0.8432515	4.6555458
H	-0.4868515	-0.8432515	4.6555458
N	0.0000000	0.0000000	4.2841757

[**2c**···N≡C–CH_3]$^-$

C	-5.2611121	3.4507053	-0.2146706
C	-6.6211291	3.0959038	-0.1900058
C	-7.0613429	1.7476672	-0.2111986
C	-6.0746273	0.7297285	-0.2426037
C	-4.7062383	1.1256839	-0.2655680
C	-4.2721513	2.4685810	-0.2639210
C	-6.0746273	-0.7297285	-0.2426037
C	-4.7062383	-1.1256839	-0.2655680
N	-3.9070147	0.0000000	-0.2948473
C	-7.0613429	-1.7476672	-0.2111986
C	-6.6211291	-3.0959038	-0.1900058
C	-5.2611121	-3.4507053	-0.2146706
C	-4.2721513	-2.4685810	-0.2639210
N	-2.9036366	-2.7255732	-0.3256467
C	-2.2840319	-3.9560593	-0.1066538

O	-2.8699545	-4.9709071	0.2428280
N	-0.9085973	-3.8774097	-0.3256467
H	-4.9816738	4.4989892	-0.1954307
H	-2.8925787	0.0000000	-0.2142950
H	-4.9816738	-4.4989892	-0.1954307
H	-2.2970548	-1.9539760	-0.6111288
H	-0.5436655	-2.9662958	-0.6111288
C	-0.3578424	-6.2816094	-0.2146706
C	0.6294332	-7.2820179	-0.1900058
C	2.0171472	-6.9891359	-0.2111986
C	2.4053502	-5.6256458	-0.2426037
C	1.3782483	-4.6385638	-0.2655680
C	-0.0017782	-4.9340820	-0.2639210
C	3.6692771	-4.8959173	-0.2426037
C	3.3279900	-3.5128799	-0.2655680
N	1.9535073	-3.3835740	-0.2948473
C	5.0441956	-5.2414687	-0.2111986
C	5.9916959	-4.1861141	-0.1900058
C	5.6189545	-2.8309041	-0.2146706
C	4.2739295	-2.4655011	-0.2639210
N	3.8122339	-1.1518365	-0.3256467
C	4.5680638	0.0000000	-0.1066538
O	5.7399091	0.0000000	0.2428280
N	3.8122339	1.1518365	-0.3256467
H	-1.4054021	-6.5637506	-0.1954307
H	1.4462894	-2.5050467	-0.2142950
H	6.3870758	-2.0647614	-0.1954307
H	2.8407202	-1.0123198	-0.6111288
H	2.8407202	1.0123198	-0.6111288
C	5.6189545	2.8309041	-0.2146706
C	5.9916959	4.1861141	-0.1900058
C	5.0441956	5.2414687	-0.2111986
C	3.6692771	4.8959173	-0.2426037
C	3.3279900	3.5128799	-0.2655680
C	4.2739295	2.4655011	-0.2639210
C	2.4053502	5.6256458	-0.2426037
C	1.3782483	4.6385638	-0.2655680
N	1.9535073	3.3835740	-0.2948473
C	2.0171472	6.9891359	-0.2111986
C	0.6294332	7.2820179	-0.1900058
C	-0.3578424	6.2816094	-0.2146706
C	-0.0017782	4.9340820	-0.2639210
N	-0.9085973	3.8774097	-0.3256467
C	-2.2840319	3.9560593	-0.1066538
O	-2.8699545	4.9709071	0.2428280
N	-2.9036366	2.7255732	-0.3256467
H	6.3870758	2.0647614	-0.1954307

H	1.4462894	2.5050467	-0.2142950
H	-1.4054021	6.5637506	-0.1954307
H	-0.5436655	2.9662958	-0.6111288
H	-2.2970548	1.9539760	-0.6111288
N	0.0000000	0.0000000	-0.9581961
O	-0.6295029	-1.0903310	-0.9612842
O	-0.6295029	1.0903310	-0.9612842
O	1.2590058	0.0000000	-0.9612842
C	-7.5760479	-4.1587347	-0.1551346
C	-8.4699647	-1.5486858	-0.2034243
C	-8.4699647	1.5486858	-0.2034243
C	-7.5760479	4.1587347	-0.1551346
C	0.1864541	8.6404173	-0.1551346
C	2.8937811	8.1095475	-0.2034243
C	5.5761836	6.5608617	-0.2034243
C	7.3895939	4.4816826	-0.1551346
C	7.3895939	-4.4816826	-0.1551346
C	5.5761836	-6.5608617	-0.2034243
C	2.8937811	-8.1095475	-0.2034243
C	0.1864541	-8.6404173	-0.1551346
N	-0.2148342	9.7339376	-0.1288349
N	3.4920379	9.1097988	-0.2004401
N	6.1432983	7.5790929	-0.2004401
N	8.5372544	4.6809169	-0.1288349
N	8.5372544	-4.6809169	-0.1288349
N	6.1432983	-7.5790929	-0.2004401
N	3.4920379	-9.1097988	-0.2004401
N	-0.2148342	-9.7339376	-0.1288349
N	-8.3224202	-5.0530207	-0.1288349
N	-9.6353361	-1.5307059	-0.2004401
N	-9.6353361	1.5307059	-0.2004401
N	-8.3224202	5.0530207	-0.1288349
N	0.0000000	0.0000000	1.9092859
C	0.0000000	0.0000000	3.0706671
C	0.0000000	0.0000000	4.5244204
H	1.0316811	0.0000000	4.9015438
H	-0.5158405	0.8934620	4.9015438
H	-0.5158405	-0.8934620	4.9015438

[**2c**···N≡CH]$^{-}$

C	-5.2658177	3.4523228	-0.0601314
C	-6.6259242	3.0977667	-0.0596855
C	-7.0654395	1.7495236	-0.0834488
C	-6.0790466	0.7301722	-0.0936348
C	-4.7105865	1.1265940	-0.0946760
C	-4.2763538	2.4704266	-0.0899990
C	-6.0790466	-0.7301722	-0.0936348

C	-4.7105865	-1.1265940	-0.0946760
N	-3.9111341	0.0000000	-0.1124873
C	-7.0654395	-1.7495236	-0.0834488
C	-6.6259242	-3.0977667	-0.0596855
C	-5.2658177	-3.4523228	-0.0601314
C	-4.2763538	-2.4704266	-0.0899990
N	-2.9073086	-2.7305230	-0.1332868
C	-2.2891604	-3.9649420	0.0744773
O	-2.8777204	-4.9843580	0.4050246
N	-0.9110480	-3.8830646	-0.1332868
H	-4.9857199	4.5006071	-0.0419109
H	-2.8991922	0.0000000	-0.0085080
H	-4.9857199	-4.5006071	-0.0419109
H	-2.2979093	-1.9562134	-0.4025271
H	-0.5451759	-2.9681545	-0.4025271
C	-0.3568904	-6.2864933	-0.0601314
C	0.6302175	-7.2871021	-0.0596855
C	2.0175878	-6.9936119	-0.0834488
C	2.4071757	-5.6296949	-0.0936348
C	1.3796342	-4.6427846	-0.0946760
C	-0.0012753	-4.9386443	-0.0899990
C	3.6718710	-4.8995227	-0.0936348
C	3.3309523	-3.5161906	-0.0946760
N	1.9555670	-3.3871415	-0.1124873
C	5.0478517	-5.2440883	-0.0834488
C	5.9957068	-4.1893354	-0.0596855
C	5.6227081	-2.8341705	-0.0601314
C	4.2776291	-2.4682177	-0.0899990
N	3.8183566	-1.1525416	-0.1332868
C	4.5783207	0.0000000	0.0744773
O	5.7554409	0.0000000	0.4050246
N	3.8183566	1.1525416	-0.1332868
H	-1.4047801	-6.5680636	-0.0419109
H	1.4495961	-2.5107741	-0.0085080
H	6.3905000	-2.0674565	-0.0419109
H	2.8430852	-1.0119411	-0.4025271
H	2.8430852	1.0119411	-0.4025271
C	5.6227081	2.8341705	-0.0601314
C	5.9957068	4.1893354	-0.0596855
C	5.0478517	5.2440883	-0.0834488
C	3.6718710	4.8995227	-0.0936348
C	3.3309523	3.5161906	-0.0946760
C	4.2776291	2.4682177	-0.0899990
C	2.4071757	5.6296949	-0.0936348
C	1.3796342	4.6427846	-0.0946760
N	1.9555670	3.3871415	-0.1124873
C	2.0175878	6.9936119	-0.0834488

C	0.6302175	7.2871021	-0.0596855
C	-0.3568904	6.2864933	-0.0601314
C	-0.0012753	4.9386443	-0.0899990
N	-0.9110480	3.8830646	-0.1332868
C	-2.2891604	3.9649420	0.0744773
O	-2.8777204	4.9843580	0.4050246
N	-2.9073086	2.7305230	-0.1332868
H	6.3905000	2.0674565	-0.0419109
H	1.4495961	2.5107741	-0.0085080
H	-1.4047801	6.5680636	-0.0419109
H	-0.5451759	2.9681545	-0.4025271
H	-2.2979093	1.9562134	-0.4025271
N	-0.0000000	0.0000000	-0.6685872
O	-0.6291849	-1.0897802	-0.6695877
O	-0.6291849	1.0897802	-0.6695877
O	1.2583698	0.0000000	-0.6695877
C	-7.5788390	-4.1633063	-0.0568785
C	-8.4735181	-1.5505293	-0.1076998
C	-8.4735181	1.5505293	-0.1076998
C	-7.5788390	4.1633063	-0.0568785
C	0.1838905	8.6451203	-0.0568785
C	2.8939613	8.1135466	-0.1076998
C	5.5795569	6.5630173	-0.1076998
C	7.3949486	4.4818140	-0.0568785
C	7.3949486	-4.4818140	-0.0568785
C	5.5795569	-6.5630173	-0.1076998
C	2.8939613	-8.1135466	-0.1076998
C	0.1838905	-8.6451203	-0.0568785
N	-0.2271895	9.7353906	-0.0633666
N	3.4984311	9.1096110	-0.1363534
N	6.1399390	7.5845357	-0.1363534
N	8.5446904	4.6709434	-0.0633666
N	8.5446904	-4.6709434	-0.0633666
N	6.1399390	-7.5845357	-0.1363534
N	3.4984311	-9.1096110	-0.1363534
N	-0.2271895	-9.7353906	-0.0633666
N	-8.3175008	-5.0644472	-0.0633666
N	-9.6383701	-1.5250753	-0.1363534
N	-9.6383701	1.5250753	-0.1363534
N	-8.3175008	5.0644472	-0.0633666
N	-0.0000000	0.0000000	2.2510614
C	-0.0000000	0.0000000	3.4079960
H	-0.0000000	0.0000000	4.4843793

[**2c**···N≡C]$^{2-}$

C	-5.2173183	3.4438824	0.0162989
C	-6.5806811	3.0993890	0.0192125
C	-7.0335110	1.7540342	0.0007836
C	-6.0541709	0.7288724	0.0090194
C	-4.6839776	1.1170603	0.0309222
C	-4.2359990	2.4505539	0.0036644
C	-6.0541709	-0.7288724	0.0090194
C	-4.6839776	-1.1170603	0.0309222
N	-3.8807986	0.0000000	0.0416514
C	-7.0335110	-1.7540342	0.0007836
C	-6.5806811	-3.0993890	0.0192125
C	-5.2173183	-3.4438824	0.0162989
C	-4.2359990	-2.4505539	0.0036644
N	-2.8681867	-2.6747225	-0.0656087
C	-2.2487245	-3.8949051	0.1704451
O	-2.8327694	-4.9065006	0.5411076
N	-0.8822843	-3.8212838	-0.0656087
H	-4.9290901	4.4896646	0.0205267
H	-2.8860241	0.0000000	0.2829256
H	-4.9290901	-4.4896646	0.0205267
H	-2.2644651	-1.8992418	-0.3696149
H	-0.5125591	-2.9107052	-0.3696149
C	-0.3738304	-6.2402714	0.0162989
C	0.6061909	-7.2487315	0.0192125
C	1.9977173	-6.9682163	0.0007836
C	2.3958634	-5.6075020	0.0090194
C	1.3745862	-4.6149737	0.0309222
C	-0.0042424	-4.8937596	0.0036644
C	3.6583075	-4.8786296	0.0090194
C	3.3093914	-3.4979134	0.0309222
N	1.9403993	-3.3608702	0.0416514
C	5.0357937	-5.2141821	0.0007836
C	5.9744901	-4.1493425	0.0192125
C	5.5911488	-2.7963890	0.0162989
C	4.2402414	-2.4432058	0.0036644
N	3.7504710	-1.1465613	-0.0656087
C	4.4974490	0.0000000	0.1704451
O	5.6655389	0.0000000	0.5411076
N	3.7504710	1.1465613	-0.0656087
H	-1.4236186	-6.5135495	0.0205267
H	1.4430121	-2.4993702	0.2829256
H	6.3527086	-2.0238849	0.0205267
H	2.7770242	-1.0114634	-0.3696149
H	2.7770242	1.0114634	-0.3696149
C	5.5911488	2.7963890	0.0162989
C	5.9744901	4.1493425	0.0192125
C	5.0357937	5.2141821	0.0007836

C	3.6583075	4.8786296	0.0090194
C	3.3093914	3.4979134	0.0309222
C	4.2402414	2.4432058	0.0036644
C	2.3958634	5.6075020	0.0090194
C	1.3745862	4.6149737	0.0309222
N	1.9403993	3.3608702	0.0416514
C	1.9977173	6.9682163	0.0007836
C	0.6061909	7.2487315	0.0192125
C	-0.3738304	6.2402714	0.0162989
C	-0.0042424	4.8937596	0.0036644
N	-0.8822843	3.8212838	-0.0656087
C	-2.2487245	3.8949051	0.1704451
O	-2.8327694	4.9065006	0.5411076
N	-2.8681867	2.6747225	-0.0656087
H	6.3527086	2.0238849	0.0205267
H	1.4430121	2.4993702	0.2829256
H	-1.4236186	6.5135495	0.0205267
H	-0.5125591	2.9107052	-0.3696149
H	-2.2644651	1.8992418	-0.3696149
N	0.0000000	0.0000000	-1.2068466
O	-0.6318991	-1.0944814	-1.2214268
O	-0.6318991	1.0944814	-1.2214268
O	1.2637983	0.0000000	-1.2214268
C	-7.5274734	-4.1700974	0.0184306
C	-8.4426595	-1.5634656	-0.0277410
C	-8.4426595	1.5634656	-0.0277410
C	-7.5274734	4.1700974	0.0184306
C	0.1523264	8.6040319	0.0184306
C	2.8673288	8.0932904	-0.0277410
C	5.5753306	6.5298248	-0.0277410
C	7.3751470	4.4339345	0.0184306
C	7.3751470	-4.4339345	0.0184306
C	5.5753306	-6.5298248	-0.0277410
C	2.8673288	-8.0932904	-0.0277410
C	0.1523264	-8.6040319	0.0184306
N	-0.2563997	9.6956587	0.0153497
N	3.4666534	9.0930526	-0.0553503
N	6.1414878	7.5487362	-0.0553503
N	8.5248866	4.6257807	0.0153497
N	8.5248866	-4.6257807	0.0153497
N	6.1414878	-7.5487362	-0.0553503
N	3.4666534	-9.0930526	-0.0553503
N	-0.2563997	-9.6956587	0.0153497
N	-8.2684869	-5.0698780	0.0153497
N	-9.6081412	-1.5443163	-0.0553503
N	-9.6081412	1.5443163	-0.0553503
N	-8.2684869	5.0698780	0.0153497

N	-0.0000000	0.0000000	1.4423685
C	0.0000000	0.0000000	2.6250103

[**2c**···Cl]$^{2-}$

C	-5.2042096	3.4418960	0.0361264
C	-6.5683207	3.1004319	0.0566892
C	-7.0239097	1.7555698	0.0517233
C	-6.0465838	0.7285926	0.0570962
C	-4.6756525	1.1146192	0.0704112
C	-4.2261764	2.4452889	0.0238722
C	-6.0465838	-0.7285926	0.0570962
C	-4.6756525	-1.1146192	0.0704112
N	-3.8699787	0.0000000	0.0833638
C	-7.0239097	-1.7555698	0.0517233
C	-6.5683207	-3.1004319	0.0566892
C	-5.2042096	-3.4418960	0.0361264
C	-4.2261764	-2.4452889	0.0238722
N	-2.8590801	-2.6615888	-0.0664966
C	-2.2360300	-3.8729176	0.1930296
O	-2.8138997	-4.8738172	0.6026263
N	-0.8754635	-3.8068304	-0.0664966
H	-4.9140774	4.4870865	0.0261030
H	-2.8751879	0.0000000	0.3427098
H	-4.9140774	-4.4870865	0.0261030
H	-2.2620971	-1.8876620	-0.3898439
H	-0.5037147	-2.9028646	-0.3898439
C	-0.3786646	-6.2279258	0.0361264
C	0.5991075	-7.2385486	0.0566892
C	1.9915868	-6.9606691	0.0517233
C	2.3923122	-5.6007915	0.0570962
C	1.3725377	-4.6065434	0.0704112
C	-0.0045941	-4.8826206	0.0238722
C	3.6542716	-4.8721988	0.0570962
C	3.3031148	-3.4919242	0.0704112
N	1.9349894	-3.3514999	0.0833638
C	5.0323229	-5.2050993	0.0517233
C	5.9692132	-4.1381166	0.0566892
C	5.5828742	-2.7860297	0.0361264
C	4.2307705	-2.4373317	0.0238722
N	3.7345436	-1.1452416	-0.0664966
C	4.4720601	0.0000000	0.1930296
O	5.6277994	0.0000000	0.6026263
N	3.7345436	1.1452416	-0.0664966
H	-1.4288922	-6.4992591	0.0261030
H	1.4375940	-2.4899858	0.3427098
H	6.3429696	-2.0121726	0.0261030
H	2.7658118	-1.0152026	-0.3898439

H	2.7658118	1.0152026	-0.3898439
C	5.5828742	2.7860297	0.0361264
C	5.9692132	4.1381166	0.0566892
C	5.0323229	5.2050993	0.0517233
C	3.6542716	4.8721988	0.0570962
C	3.3031148	3.4919242	0.0704112
C	4.2307705	2.4373317	0.0238722
C	2.3923122	5.6007915	0.0570962
C	1.3725377	4.6065434	0.0704112
N	1.9349894	3.3514999	0.0833638
C	1.9915868	6.9606691	0.0517233
C	0.5991075	7.2385486	0.0566892
C	-0.3786646	6.2279258	0.0361264
C	-0.0045941	4.8826206	0.0238722
N	-0.8754635	3.8068304	-0.0664966
C	-2.2360300	3.8729176	0.1930296
O	-2.8138997	4.8738172	0.6026263
N	-2.8590801	2.6615888	-0.0664966
H	6.3429696	2.0121726	0.0261030
H	1.4375940	2.4899858	0.3427098
H	-1.4288922	6.4992591	0.0261030
H	-0.5037147	2.9028646	-0.3898439
H	-2.2620971	1.8876620	-0.3898439
N	0.0000000	0.0000000	-1.3739646
O	-0.6329256	-1.0962593	-1.3852370
O	-0.6329256	1.0962593	-1.3852370
O	1.2658512	0.0000000	-1.3852370
C	-7.5130989	-4.1728383	0.0579721
C	-8.4334930	-1.5664024	0.0404342
C	-8.4334930	1.5664024	0.0404342
C	-7.5130989	4.1728383	0.0579721
C	0.1427655	8.5929537	0.0579721
C	2.8602023	8.0868204	0.0404342
C	5.5732908	6.5204180	0.0404342
C	7.3703334	4.4201154	0.0579721
C	7.3703334	-4.4201154	0.0579721
C	5.5732908	-6.5204180	0.0404342
C	2.8602023	-8.0868204	0.0404342
C	0.1427655	-8.5929537	0.0579721
N	-0.2679617	9.6838870	0.0565344
N	3.4603851	9.0864314	0.0286493
N	6.1388879	7.5399971	0.0286493
N	8.5204730	4.6098818	0.0565344
N	8.5204730	-4.6098818	0.0565344
N	6.1388879	-7.5399971	0.0286493
N	3.4603851	-9.0864314	0.0286493
N	-0.2679617	-9.6838870	0.0565344

N	-8.2525113	-5.0740052	0.0565344
N	-9.5992730	-1.5464343	0.0286493
N	-9.5992730	1.5464343	0.0286493
N	-8.2525113	5.0740052	0.0565344
Cl	-0.0000000	0.0000000	1.5688580

[**2c**···BF_4]$^{2-}$

C	-5.2558219	3.4467465	-0.0735663
C	-6.6135154	3.0917233	-0.1398099
C	-7.0520668	1.7441819	-0.1910906
C	-6.0625640	0.7286061	-0.1704087
C	-4.6936329	1.1230284	-0.1268793
C	-4.2610674	2.4662903	-0.0710885
C	-6.0625640	-0.7286061	-0.1704087
C	-4.6936329	-1.1230284	-0.1268793
N	-3.8947974	0.0000000	-0.1104971
C	-7.0520668	-1.7441819	-0.1910906
C	-6.6135154	-3.0917233	-0.1398099
C	-5.2558219	-3.4467465	-0.0735663
C	-4.2610674	-2.4662903	-0.0710885
N	-2.8987297	-2.7250942	-0.0085920
C	-2.2979477	-3.9801621	-0.0393097
O	-2.9097237	-5.0397892	-0.0946126
N	-0.9106360	-3.8729207	-0.0085920
H	-4.9777756	4.4939517	-0.0249228
H	-2.8794734	0.0000000	-0.2105117
H	-4.9777756	-4.4939517	-0.0249228
H	-2.2613218	-1.9252985	-0.0045227
H	-0.5366965	-2.9210114	-0.0045227
C	-0.3570591	-6.2750485	-0.0735663
C	0.6292468	-7.2733339	-0.1398099
C	2.0155276	-6.9793599	-0.1910906
C	2.4002906	-5.6146375	-0.1704087
C	1.3742454	-4.6263195	-0.1268793
C	-0.0053364	-4.9233378	-0.0710885
C	3.6622734	-4.8860314	-0.1704087
C	3.3193875	-3.5032912	-0.1268793
N	1.9473987	-3.3729935	-0.1104971
C	5.0365392	-5.2351780	-0.1910906
C	5.9842686	-4.1816107	-0.1398099
C	5.6128810	-2.8283021	-0.0735663
C	4.2664038	-2.4570474	-0.0710885
N	3.8093657	-1.1478265	-0.0085920
C	4.5958954	0.0000000	-0.0393097
O	5.8194473	0.0000000	-0.0946126
N	3.8093657	1.1478265	-0.0085920
H	-1.4029885	-6.5578560	-0.0249228

H	1.4397367	-2.4936971	-0.2105117
H	6.3807641	-2.0639043	-0.0249228
H	2.7980183	-0.9957129	-0.0045227
H	2.7980183	0.9957129	-0.0045227
C	5.6128810	2.8283021	-0.0735663
C	5.9842686	4.1816107	-0.1398099
C	5.0365392	5.2351780	-0.1910906
C	3.6622734	4.8860314	-0.1704087
C	3.3193875	3.5032912	-0.1268793
C	4.2664038	2.4570474	-0.0710885
C	2.4002906	5.6146375	-0.1704087
C	1.3742454	4.6263195	-0.1268793
N	1.9473987	3.3729935	-0.1104971
C	2.0155276	6.9793599	-0.1910906
C	0.6292468	7.2733339	-0.1398099
C	-0.3570591	6.2750485	-0.0735663
C	-0.0053364	4.9233378	-0.0710885
N	-0.9106360	3.8729207	-0.0085920
C	-2.2979477	3.9801621	-0.0393097
O	-2.9097237	5.0397892	-0.0946126
N	-2.8987297	2.7250942	-0.0085920
H	6.3807641	2.0639043	-0.0249228
H	1.4397367	2.4936971	-0.2105117
H	-1.4029885	6.5578560	-0.0249228
H	-0.5366965	2.9210114	-0.0045227
H	-2.2613218	1.9252985	-0.0045227
N	0.0000000	0.0000000	-0.8367074
O	-0.6311495	-1.0931829	-0.8546649
O	-0.6311495	1.0931829	-0.8546649
O	1.2622989	0.0000000	-0.8546649
C	-7.5685967	-4.1549891	-0.1524796
C	-8.4583515	-1.5479856	-0.2729997
C	-8.4583515	1.5479856	-0.2729997
C	-7.5685967	4.1549891	-0.1524796
C	0.1859722	8.6320915	-0.1524796
C	2.8885809	8.0991401	-0.2729997
C	5.5697706	6.5511545	-0.2729997
C	7.3826244	4.4771024	-0.1524796
C	7.3826244	-4.4771024	-0.1524796
C	5.5697706	-6.5511545	-0.2729997
C	2.8885809	-8.0991401	-0.2729997
C	0.1859722	-8.6320915	-0.1524796
N	-0.2139175	9.7268046	-0.1619830
N	3.4809608	9.1003674	-0.3503783
N	6.1406690	7.5647842	-0.3503783
N	8.5306186	4.6781443	-0.1619830
N	8.5306186	-4.6781443	-0.1619830

N	6.1406690	-7.5647842	-0.3503783
N	3.4809608	-9.1003674	-0.3503783
N	-0.2139175	-9.7268046	-0.1619830
N	-8.3167011	-5.0486602	-0.1619830
N	-9.6216298	-1.5355833	-0.3503783
N	-9.6216298	1.5355833	-0.3503783
N	-8.3167011	5.0486602	-0.1619830
B	0.0000000	0.0000000	3.0828549
F	1.3435713	0.0000000	3.5133154
F	-0.6717857	1.1635669	3.5133154
F	-0.6717857	-1.1635669	3.5133154
F	0.0000000	0.0000000	1.6350198

[**2a**···N≡C]$^{2-}$

C	5.3168945	-3.4309322	-0.0108620
C	6.6669870	-3.0320145	0.0073225
C	7.0413116	-1.6912353	0.0042655
C	6.0250735	-0.7228125	-0.0004905
C	4.6643882	-1.1257221	-0.0047629
C	4.2820114	-2.4822474	-0.0264576
C	6.0250735	0.7228125	-0.0004905
C	4.6643882	1.1257221	-0.0047629
N	3.8533535	0.0000000	-0.0198617
C	7.0413116	1.6912353	0.0042655
C	6.6669870	3.0320145	0.0073225
C	5.3168945	3.4309322	-0.0108620
C	4.2820114	2.4822474	-0.0264576
N	2.9174898	2.7682129	-0.0839389
C	2.3149087	4.0095396	0.0545358
O	2.9215498	5.0602726	0.2695973
N	0.9385978	3.9107267	-0.0839389
H	5.0520165	-4.4838763	-0.0101301
H	7.4345812	-3.8087562	0.0181279
H	8.0916636	-1.3954318	0.0110623
H	2.8488746	0.0000000	0.1646402
H	8.0916636	1.3954318	0.0110623
H	7.4345812	3.8087562	0.0181279
H	5.0520165	4.4838763	-0.0101301
H	2.2883740	1.9809977	-0.2730853
H	0.5714074	2.9722889	-0.2730853
C	0.3128272	6.3200318	-0.0108620
C	-0.7076919	7.2897874	0.0073225
C	-2.0560030	6.9435724	0.0042655
C	-2.3865628	5.5792729	-0.0004905
C	-1.3572901	4.6023398	-0.0047629
C	0.0086836	4.9494544	-0.0264576
C	-3.6385107	4.8564604	-0.0004905

C	-3.3070981	3.4766176	-0.0047629
N	-1.9266768	3.3371020	-0.0198617
C	-4.9853085	5.2523370	0.0042655
C	-5.9592951	4.2577729	0.0073225
C	-5.6297217	2.8890996	-0.0108620
C	-4.2906950	2.4672069	-0.0264576
N	-3.8560876	1.1425138	-0.0839389
C	-4.6298175	0.0000000	0.0545358
O	-5.8430995	0.0000000	0.2695973
N	-3.8560876	-1.1425138	-0.0839389
H	1.3571425	6.6171127	-0.0101301
H	-0.4188110	8.3429143	0.0181279
H	-2.8373524	7.7053021	0.0110623
H	-1.4244373	2.4671978	0.1646402
H	-5.2543112	6.3098703	0.0110623
H	-7.0157702	4.5341581	0.0181279
H	-6.4091590	2.1332365	-0.0101301
H	-2.8597813	0.9912911	-0.2730853
H	-2.8597813	-0.9912911	-0.2730853
C	-5.6297217	-2.8890996	-0.0108620
C	-5.9592951	-4.2577729	0.0073225
C	-4.9853085	-5.2523370	0.0042655
C	-3.6385107	-4.8564604	-0.0004905
C	-3.3070981	-3.4766176	-0.0047629
C	-4.2906950	-2.4672069	-0.0264576
C	-2.3865628	-5.5792729	-0.0004905
C	-1.3572901	-4.6023398	-0.0047629
N	-1.9266768	-3.3371020	-0.0198617
C	-2.0560030	-6.9435724	0.0042655
C	-0.7076919	-7.2897874	0.0073225
C	0.3128272	-6.3200318	-0.0108620
C	0.0086836	-4.9494544	-0.0264576
N	0.9385978	-3.9107267	-0.0839389
C	2.3149087	-4.0095396	0.0545358
O	2.9215498	-5.0602726	0.2695973
N	2.9174898	-2.7682129	-0.0839389
H	-6.4091590	-2.1332365	-0.0101301
H	-7.0157702	-4.5341581	0.0181279
H	-5.2543112	-6.3098703	0.0110623
H	-1.4244373	-2.4671978	0.1646402
H	-2.8373524	-7.7053021	0.0110623
H	-0.4188110	-8.3429143	0.0181279
H	1.3571425	-6.6171127	-0.0101301
H	0.5714074	-2.9722889	-0.2730853
H	2.2883740	-1.9809977	-0.2730853
N	-0.0000000	0.0000000	-0.9633341
O	0.6314744	1.0937457	-0.9803762

O	0.6314744	-1.0937457	-0.9803762
O	-1.2629487	0.0000000	-0.9803762
N	0.0000000	0.0000000	1.7646490
C	0.0000000	0.0000000	2.9467721

[**2a**···Cl]$^{2-}$

C	5.2933116	-3.4280055	0.0381992
C	6.6437275	-3.0356807	0.1066072
C	7.0239370	-1.6960022	0.1261869
C	6.0127315	-0.7227945	0.1008350
C	4.6514049	-1.1216761	0.0610498
C	4.2652258	-2.4726165	0.0013873
C	6.0127315	0.7227945	0.1008350
C	4.6514049	1.1216761	0.0610498
N	3.8365368	0.0000000	0.0340725
C	7.0239370	1.6960022	0.1261869
C	6.6437275	3.0356807	0.1066072
C	5.2933116	3.4280055	0.0381992
C	4.2652258	2.4726165	0.0013873
N	2.9032498	2.7448374	-0.1266534
C	2.2885779	3.9639333	0.1005728
O	2.8783921	4.9855214	0.4587348
N	0.9254741	3.8867068	-0.1266534
H	5.0248133	-4.4798329	0.0153858
H	7.4077688	-3.8156976	0.1325939
H	8.0753538	-1.4057996	0.1629947
H	2.8369195	0.0000000	0.2669628
H	8.0753538	1.4057996	0.1629947
H	7.4077688	3.8156976	0.1325939
H	5.0248133	4.4798329	0.0153858
H	2.2937054	1.9667710	-0.4056502
H	0.5564210	2.9697927	-0.4056502
C	0.3220840	6.2981451	0.0381992
C	-0.6928871	7.2714772	0.1066072
C	-2.0431876	6.9309090	0.1261869
C	-2.3804073	5.5685755	0.1008350
C	-1.3543025	4.5890729	0.0610498
C	0.0087358	4.9301022	0.0013873
C	-3.6323242	4.8457810	0.1008350
C	-3.2971024	3.4673968	0.0610498
N	-1.9182684	3.3225384	0.0340725
C	-4.9807495	5.2349068	0.1261869
C	-5.9508404	4.2357965	0.1066072
C	-5.6153957	2.8701396	0.0381992
C	-4.2739616	2.4574856	0.0013873
N	-3.8287239	1.1418694	-0.1266534
C	-4.5771559	0.0000000	0.1005728

O	-5.7567843	0.0000000	0.4587348
N	-3.8287239	-1.1418694	-0.1266534
H	1.3672424	6.5915324	0.0153858
H	-0.3993934	8.3231648	0.1325939
H	-2.8202187	7.6963614	0.1629947
H	-1.4184598	2.4568444	0.2669628
H	-5.2551351	6.2905618	0.1629947
H	-7.0083755	4.5074672	0.1325939
H	-6.3920557	2.1116995	0.0153858
H	-2.8501264	1.0030217	-0.4056502
H	-2.8501264	-1.0030217	-0.4056502
C	-5.6153957	-2.8701396	0.0381992
C	-5.9508404	-4.2357965	0.1066072
C	-4.9807495	-5.2349068	0.1261869
C	-3.6323242	-4.8457810	0.1008350
C	-3.2971024	-3.4673968	0.0610498
C	-4.2739616	-2.4574856	0.0013873
C	-2.3804073	-5.5685755	0.1008350
C	-1.3543025	-4.5890729	0.0610498
N	-1.9182684	-3.3225384	0.0340725
C	-2.0431876	-6.9309090	0.1261869
C	-0.6928871	-7.2714772	0.1066072
C	0.3220840	-6.2981451	0.0381992
C	0.0087358	-4.9301022	0.0013873
N	0.9254741	-3.8867068	-0.1266534
C	2.2885779	-3.9639333	0.1005728
O	2.8783921	-4.9855214	0.4587348
N	2.9032498	-2.7448374	-0.1266534
H	-6.3920557	-2.1116995	0.0153858
H	-7.0083755	-4.5074672	0.1325939
H	-5.2551351	-6.2905618	0.1629947
H	-1.4184598	-2.4568444	0.2669628
H	-2.8202187	-7.6963614	0.1629947
H	-0.3993934	-8.3231648	0.1325939
H	1.3672424	-6.5915324	0.0153858
H	0.5564210	-2.9697927	-0.4056502
H	2.2937054	-1.9667710	-0.4056502
N	0.0000000	0.0000000	-1.3632214
O	0.6331236	1.0966023	-1.3763713
O	0.6331236	-1.0966023	-1.3763713
O	-1.2662473	0.0000000	-1.3763713
Cl	-0.0000000	0.0000000	1.6336888

[**2a**···BF_4]$^{2-}$

C	-5.3372935	3.4331985	-0.1625430
C	-6.6854171	3.0283598	-0.1660289
C	-7.0525071	1.6861053	-0.1775403

C	-6.0315562	0.7228163	-0.1779596
C	-4.6719432	1.1295249	-0.1725583
C	-4.2968088	2.4909868	-0.1696040
C	-6.0315562	-0.7228163	-0.1779596
C	-4.6719432	-1.1295249	-0.1725583
N	-3.8633198	0.0000000	-0.1882881
C	-7.0525071	-1.6861053	-0.1775403
C	-6.6854171	-3.0283598	-0.1660289
C	-5.3372935	-3.4331985	-0.1625430
C	-4.2968088	-2.4909868	-0.1696040
N	-2.9336606	-2.7936869	-0.1821879
C	-2.3363075	-4.0466033	-0.1233067
O	-2.9504289	-5.1102928	-0.0378336
N	-0.9525735	-3.9374680	-0.1821879
H	-5.0768190	4.4871964	-0.1491771
H	-7.4565559	3.8014114	-0.1588062
H	-8.1010561	1.3843591	-0.1789835
H	-2.8517569	0.0000000	-0.0678893
H	-8.1010561	-1.3843591	-0.1789835
H	-7.4565559	-3.8014114	-0.1588062
H	-5.0768190	-4.4871964	-0.1491771
H	-2.2937395	-2.0012225	-0.2689990
H	-0.5862398	-2.9870480	-0.2689990
C	-0.3045903	-6.3388310	-0.1625430
C	0.7200720	-7.3039209	-0.1660289
C	2.0660435	-6.9507029	-0.1775403
C	2.3898009	-5.5848890	-0.1779596
C	1.3577743	-4.6107839	-0.1725583
C	-0.0088534	-4.9666389	-0.1696040
C	3.6417553	-4.8620728	-0.1779596
C	3.3141689	-3.4812590	-0.1725583
N	1.9316599	-3.3457331	-0.1882881
C	4.9864635	-5.2645976	-0.1775403
C	5.9653451	-4.2755611	-0.1660289
C	5.6418839	-2.9056325	-0.1625430
C	4.3056622	-2.4756522	-0.1696040
N	3.8862341	-1.1437812	-0.1821879
C	4.6726150	0.0000000	-0.1233067
O	5.9008578	0.0000000	-0.0378336
N	3.8862341	1.1437812	-0.1821879
H	-1.3476165	-6.6402524	-0.1491771
H	0.4361591	-8.3582725	-0.1588062
H	2.8516380	-7.7078999	-0.1789835
H	1.4258785	-2.4696939	-0.0678893
H	5.2494182	-6.3235409	-0.1789835
H	7.0203968	-4.5568611	-0.1588062
H	6.4244356	-2.1530561	-0.1491771

H	2.8799793	-0.9858254	-0.2689990
H	2.8799793	0.9858254	-0.2689990
C	5.6418839	2.9056325	-0.1625430
C	5.9653451	4.2755611	-0.1660289
C	4.9864635	5.2645976	-0.1775403
C	3.6417553	4.8620728	-0.1779596
C	3.3141689	3.4812590	-0.1725583
C	4.3056622	2.4756522	-0.1696040
C	2.3898009	5.5848890	-0.1779596
C	1.3577743	4.6107839	-0.1725583
N	1.9316599	3.3457331	-0.1882881
C	2.0660435	6.9507029	-0.1775403
C	0.7200720	7.3039209	-0.1660289
C	-0.3045903	6.3388310	-0.1625430
C	-0.0088534	4.9666389	-0.1696040
N	-0.9525735	3.9374680	-0.1821879
C	-2.3363075	4.0466033	-0.1233067
O	-2.9504289	5.1102928	-0.0378336
N	-2.9336606	2.7936869	-0.1821879
H	6.4244356	2.1530561	-0.1491771
H	7.0203968	4.5568611	-0.1588062
H	5.2494182	6.3235409	-0.1789835
H	1.4258785	2.4696939	-0.0678893
H	2.8516380	7.7078999	-0.1789835
H	0.4361591	8.3582725	-0.1588062
H	-1.3476165	6.6402524	-0.1491771
H	-0.5862398	2.9870480	-0.2689990
H	-2.2937395	2.0012225	-0.2689990
N	0.0000000	0.0000000	-0.7821148
O	-0.6307536	-1.0924972	-0.7994709
O	-0.6307536	1.0924972	-0.7994709
O	1.2615071	0.0000000	-0.7994709
F	1.3417083	0.0000000	3.7121071
F	-0.6708542	1.1619535	3.7121071
F	-0.6708542	-1.1619535	3.7121071
F	0.0000000	0.0000000	1.8216976
B	-0.0000000	0.0000000	3.2607830

EVIKEA (selected fragments)

N	2.44500000	5.65400000	4.60400000
O	3.09600000	4.61900000	4.76100000
O	2.84000000	6.74600000	5.06900000
O	1.36000000	5.64500000	3.96800000
Br	3.75500000	6.74500000	1.69000000
N	3.92500000	2.42200000	-2.79800000
H	3.53700000	3.18300000	-2.48500000

N	7.97700000	4.09900000	-1.62100000
H	8.79500000	4.28300000	-1.81500000
C	4.50300000	2.73100000	-4.13700000
H	3.80100000	3.00200000	-4.73400000
H	4.93500000	1.94800000	-4.48700000
H	5.14400000	3.44000000	-4.05300000
C	2.85800000	1.38600000	-2.91200000
H	2.19700000	1.67600000	-3.54400000
H	2.44600000	1.25600000	-2.05400000
H	3.24600000	0.56000000	-3.20900000
C	4.97800000	1.99500000	-1.78800000
H	4.54200000	1.79500000	-0.94400000
H	5.40400000	1.18100000	-2.10000000
C	6.02100000	3.02600000	-1.55900000
C	7.30400000	3.01700000	-2.05300000
H	7.65500000	2.35800000	-2.60700000
C	7.16000000	4.86500000	-0.82200000
C	7.39200000	6.08000000	-0.19200000
H	8.21400000	6.50800000	-0.26800000
C	6.36900000	6.63900000	0.55300000
H	6.49700000	7.45000000	0.98900000
C	5.13200000	5.96600000	0.64500000
C	4.87900000	4.76500000	0.01600000
H	4.05700000	4.33900000	0.09900000
C	5.91300000	4.21200000	-0.75400000

ORUHOZ (selected fragments)

O	4.24700000	1.83700000	10.60000000
O	4.35800000	2.86100000	8.69500000
O	6.16700000	2.49600000	9.83400000
N	4.92500000	2.39000000	9.71700000
Ag	6.34000000	5.40300000	15.93400000
Cl	4.37600000	5.40600000	10.48900000
Cl	3.98400000	8.63400000	11.72400000
Cl	9.02800000	2.41300000	20.13200000
Cl	8.77300000	5.69900000	21.18300000
N	5.30100000	5.25800000	13.01900000
N	5.02400000	7.24000000	13.81800000
N	7.76200000	3.66500000	18.08400000
N	7.60600000	5.70800000	18.72900000
C	5.47600000	6.00400000	14.14100000
C	4.75100000	6.02300000	12.01900000
C	4.59200000	7.25400000	12.50700000
C	5.68600000	3.84800000	12.90400000
H	6.18200000	3.57800000	13.70400000
H	4.88100000	3.29600000	12.81600000
H	6.25200000	3.72900000	12.11300000
C	4.98900000	8.38000000	14.72800000
H	5.35900000	8.11700000	15.59600000
H	5.52300000	9.11200000	14.35500000
H	4.06300000	8.67800000	14.84300000
C	7.30900000	4.87600000	17.70100000
C	8.34800000	3.74400000	19.31900000
C	8.25000000	5.01000000	19.72900000
C	7.72500000	2.46100000	17.24700000
H	7.18100000	2.63300000	16.45000000
H	8.63700000	2.22300000	16.97600000
H	7.33300000	1.72100000	17.75700000
C	7.33000000	7.15100000	18.73200000
H	6.42300000	7.31000000	18.39700000
H	7.40700000	7.49800000	19.64500000
H	7.97600000	7.60800000	18.15300000

BIDHAX (selected fragments)

S	4.41300000	1.17300000	7.68600000
C	3.51700000	2.31400000	6.64300000
N	2.51500000	3.02500000	7.07300000
N	3.95400000	2.36900000	5.40900000
H	2.27800000	2.99400000	7.90700000
H	2.09000000	3.58600000	6.55100000
H	3.50100000	2.87200000	4.77400000
H	4.60100000	1.89000000	5.24100000
S	3.34600000	1.17300000	9.40300000
C	4.24200000	2.31400000	10.44600000
N	5.24400000	3.02500000	10.01600000
N	3.80600000	2.36900000	11.68000000
H	5.48100000	2.99400000	9.18200000
H	5.66900000	3.58600000	10.53800000
H	4.25800000	2.87200000	12.31500000
H	3.15800000	1.89000000	11.84800000
N	1.90900000	-0.93500000	7.10000000
O	2.43500000	-1.77300000	7.83900000
O	0.90700000	-0.27200000	7.48500000
O	2.37600000	-0.71600000	5.94800000
N	6.30700000	4.99200000	12.79600000
O	6.83300000	5.82900000	13.53600000
O	5.30500000	4.32800000	13.18100000
O	6.77400000	4.77200000	11.64400000

3EZH (selected fragments)

C	0.75558200	-3.02419100	-1.39638900
C	-0.09398300	-2.99698400	-0.14907800
O	0.38428300	-2.71896600	0.95068300
N	-1.38596700	-3.28989600	-0.27744200
C	-2.22249700	-3.23859200	0.90758500
N	3.35037000	-1.30083100	0.58474500
C	3.71711400	-0.63272700	-0.51014400
N	4.99608300	-0.64909100	-0.95998300
N	2.77046700	0.04515400	-1.17086900
C	-0.68347700	2.93629700	-1.39494800
C	0.18664000	3.00036100	-0.14621000
O	-0.27378000	2.73287200	0.94135200
N	1.45956700	3.34130500	-0.30619800
C	2.32096400	3.35489000	0.86929400
N	-3.42488200	1.31006300	0.59313300
C	-3.80710900	0.64032100	-0.49578500
N	-5.09696100	0.72725200	-0.94499700

N	-2.89038500	-0.08484500	-1.15927200
N	0.02937300	-0.04981600	1.13379500
O	-1.01282100	-0.21075100	1.79668600
O	1.01583200	0.20171900	1.75564000
O	-0.03678600	-0.13447700	-0.09291300
H	1.58348600	-2.37178800	-1.21241400
H	0.92733500	-4.05580600	-1.62259700
H	-2.88811300	-2.41945100	0.73190500
H	2.39162700	-1.24062700	0.86256900
H	5.24290800	-0.13886000	-1.78384100
H	5.69254100	-1.17139200	-0.46789700
H	3.00671400	0.55816300	-1.99610200
H	1.82710200	0.03850300	-0.83917900
H	-0.03711200	2.68308300	-2.20919300
H	-1.51726500	2.30872500	-1.15862100
H	1.78048000	2.82764900	1.62744600
H	-2.47849300	1.20985800	0.90022900
H	-3.15354400	-0.59511400	-1.97803500
H	-1.94516300	-0.11411600	-0.83415600
H	-5.37704100	0.22279000	-1.76174300
H	-5.75934200	1.29523200	-0.45647900
H	2.50707500	4.33020300	1.26807700
H	3.24455500	2.85512900	0.66406600
H	3.96026300	-1.85720700	1.14908200
H	0.22851500	-2.61507800	-2.23288600
H	-1.69559700	-3.01320000	1.81117500
H	-2.78634800	-4.13961200	1.03060200
H	-1.07820900	3.90846200	-1.60466700
H	-4.03072200	1.90647700	1.11967600
H	-1.77007200	-3.53867200	-1.16658400
H	1.81752000	3.58215700	-1.20834000
